# Surface Microbiology of the iPad Tablet Computer and the Potential to Serve as a Fomite in Both Inpatient Practice Settings as Well as Outside of the Hospital Environment

**DOI:** 10.1371/journal.pone.0111250

**Published:** 2014-10-31

**Authors:** Elizabeth B. Hirsch, Brian R. Raux, Jason W. Lancaster, Rachael L. Mann, Steven N. Leonard

**Affiliations:** Northeastern University, Bouvé College of Health Sciences, School of Pharmacy, Boston, Massachusetts, United States of America; Kent State University, United States of America

## Abstract

**Background:**

The use of tablet computers and other touch screen technology within the healthcare system has rapidly expanded. It has been reported that these devices can harbor pathogens in hospitals; however, much less is known about what pathogens they can harbor when used outside the hospital environment compared to hospital practice.

**Methods:**

Thirty iPads belonging to faculty with a variety of practice settings were sampled to determine the presence and quantity of clinically-relevant organisms. Flocked nylon swabs and neutralizer solution were used to sample the surface of each iPad. Samples were then plated on a variety of selective agars for presence and quantity of selected pathogens. In addition, faculty members were surveyed to classify the physical location of their practice settings and usage patterns. Continuous variables were compared via an unpaired Student's *t* test with two-tailed distribution; categorical variables were compared with the Fisher's exact test.

**Results:**

Of the iPads sampled, 16 belonged to faculty practicing within a hospital and 14 belonged to a faculty member practicing outside a hospital. More faculty within the hospital group used their iPads at their practice sites (78.6% vs. 31.3%; p = 0.014) and within patient care areas (71.4% vs. 18.8%; p = 0.009) than the non-hospital group. There were no differences in the presence, absence, or quantity of, any of the pathogens selectively isolated between groups. Problematic nosocomial pathogens such as methicillin-resistant *Staphylococcus aureus* (MRSA), vancomycin-resistant enterococci (VRE), and *P. aeruginosa* were isolated from both hospital and non-hospital faculty iPads.

**Conclusions:**

Gram positive and Gram negative organisms were recovered from the surfaces of iPads regardless of practice setting; these included problematic multidrug-resistant pathogens like MRSA, VRE, and *Pseudomonas aeruginosa*. Healthcare personnel in all settings should be aware of the potential for tablet computers to serve as a nidus for microorganism transmission.

## Introduction

Many hospital-associated pathogens are capable of surviving on environmental surfaces and these surfaces, therefore, are a potential reservoir for transmission of infection. For this reason, many surfaces within the healthcare setting have been examined for the presence of microorganisms [1], [2], [3], [4], [5]. Specifically, handheld devices and other similar technologies have previously been shown to act as fomites [2],[3],[6],[7],[8],[9]. Recently, the use of tablet computers (such as the Apple iPad [Apple Corp., Cupertino, CA]) within the healthcare system has rapidly expanded; their use may span multiple functions, including patient interaction and education, study consent, medical applications (“apps”), or sharing of data between clinicians [10],[11],[12]. A few studies have assessed the ability of tablets to serve as fomites and found that they, like other environmental surfaces in the healthcare system, are capable of acting as reservoirs for potentially infectious agents [13],[14]. However, little is known about the potential of these devices to harbor pathogens in non-hospital healthcare settings. The objective of this study was to elucidate the presence of microorganisms on iPads used by clinicians in a variety of settings under conditions of normal usage and to compare species prevalence by type of practice site.

## Methods

This study was conducted at Northeastern University in Boston, MA, USA. Approximately 6 months following uniform distribution of iPads to all Department of Pharmacy Practice faculty with a variety of practice settings the iPad's screens were sampled to determine the presence, and quantity (if present in great enough numbers), of clinically-relevant organisms. All faculty iPads (n = 30; second-generation model) were sampled as a part of this study. The faculty within the department were categorized as having physical practice setting locations as either hospital or non-hospital (e.g., ambulatory care clinics or purely academic) sites. Faculty were not given any specific instructions on how to use their iPads, how or when to wash their hands, how or when to use alcohol sanitizing gel, how or when to clean the surface of the device, or any other instruction which might affect the results as the goal was to determine what was present under conditions of normal usage. All sampling of the iPads was performed on days faculty were on campus and not at their practice site.

Neutralizer (polysorbate 80 30 g/L, saponin 30 g/L, lecithin 3 g/L, pH 7) solution and flocked nylon swabs were used for screen sampling based on a modified protocol published by Hedin et al [15]. As the surface area of the iPad screen was much larger than the 5×5 cm testing surface used in their methods, we tested the use of 2, 3 and 4 wet swabs (plus 1 dry) using a known inoculum of several different organisms to determine how to best modify the swab count (Table S1). We deemed the use of more than 5 swabs to be impractical; therefore, five (4 wet, 1 dry) swabs were used to sample each iPad screen For each iPad, the 5 swabs were immersed in 3 mL neutralizer, vortexed, serially diluted, and plated on selective media. In addition to quantification, and because of yield and quantification limits of detection, all samples were inoculated into Mueller Hinton broth, incubated overnight, and then plated on selective media to determine pathogen presence or absence. Plates were incubated at 35°C for 24 hours prior to counting. No other parts of the iPads were screened as covers were not standardized among the groups.

Selective agars utilized for Gram positive organism quantification included mannitol salt (Staphylococci), bile esculin azide (Enteroccocci), bile esculin azide +6 mg/L vancomycin (vancomycin-resistant Enterococci; VRE), and BBL CHROMagar MRSA II (Becton, Dickinson, and Company; Sparks, MD; methicillin-resistant *Staphylococcus aureus* [MRSA]). Gram negative organism quantification was performed with BBL Levine EMB (Becton, Dickinson, and Company; Sparks, MD; Gram negative enteric organisms), MacConkey (lactose fermenting Enterobacteriaceae), and cetrimide (*Pseudomonas aeruginosa*) agars.

Alongside the iPad sampling, faculty members answered an electronic survey to classify the physical location of their practice settings (hospital or non-hospital) and to categorize the overall frequency of use, use in patient care areas, use of device covers, and cleaning habits.

### Ethics statement

This study was reviewed by the Northeastern University institutional review board and deemed exempt. The decision to participate in the survey and microbial sampling was solely that of the individual faculty and entirely voluntary.

### Statistical analysis

All statistical analyses were performed using Systat version 13.0 (Systat Software, Chicago, IL). All continuous variables were compared via an unpaired Student's *t* test with two-tailed distribution; categorical variables were compared with the Fisher's exact test. Data were expressed as mean ± standard deviation (SD) unless otherwise noted. *P* values ≤0.05 were considered statistically significant.

## Results

Thirty iPads were swabbed from pharmacy faculty with practice sites located physically in hospital (n = 14) and non-hospital (n = 16) settings. Faculty practicing within non-hospital settings included those with outpatient ambulatory care clinics (n = 7) or purely academic positions with no clinical practice (n = 9). There were no differences in cleaning or usage, however, more faculty within the hospital group used their iPads at their practice sites (78.6% vs. 31.3%; p = 0.014) and within patient care areas (71.4% vs. 18.8%; p = 0.009) than the non-hospital group (Table 1).

**Table 1:** Faculty iPad usage and care assessed via an electronic survey.

	Hospital location (n = 14)	Non-hospital location (n = 16)	*P* value
**Total frequency of use, n (%)**			
Daily	8 (57.1)	12 (75)	0.442
Weekly	5 (35.7)	3 (18.8)	0.417
**Use at practice site, n (%)**	11 (78.6)	5 (31.3)	0.014
Daily	6 (42.9)	2 (12.5)	0.092
Weekly	3 (21.4)	3 (18.8)	1.000
**Use in patient care area, n (%)**	10 (71.4)	3 (18.8)	0.009
Daily	4 (28.6)	1 (6.3)	0.157
Weekly	5 (35.7)	1 (6.3)	0.072
**Cover on device, n (%)**			
Cover on front only	3 (21.4)	3 (18.8)	1.000
Cover on back only	0 (0)	1 (6.3)	1.000
Cover on both front and back	10 (71.4)	11 (68.8)	1.000
Screen protector in use	11 (78.6)	8 (50)	0.142
**Cleaning habits, n (%)**			
Cleaned iPad	6 (42.9)	9 (56.3)	0.715
Cleaned weekly	3 (21.4)	5 (31.3)	0.689
Cleaned monthly	2 (14.3)	3 (18.8)	1.000
Cleaned with cloth only	2 (14.3)	3 (18.8)	1.000
Cleaned with alcohol only	3 (21.4)	2 (12.5)	0.642
Cleaned with alcohol and cloth	1 (7.1)	3 (18.8)	0.602
Cleaned with other	0	1 (6.3)	1.000

Overall, Gram positive organisms were more frequently recovered than Gram negative organisms (Table 2). There were no differences in the presence, absence, or quantity of, any of the pathogens selectively isolated between groups. Mean organism recovery tended to be low, ranging from 1–3 log colony forming units (CFU)/mL, however, from preliminary yield testing, it was known that mean yield from the screens was generally low, so the actual number of organisms on the screens was likely greater (Table S1). Common skin flora such as coagulase negative Staphylococci were collected from the vast majority of iPads. Problematic nosocomial pathogens such as MRSA (64.3% and 37.5%), VRE (7.1% and 0%), and *P. aeruginosa* (7.1% and 6.3%) were isolated from both hospital and non-hospital faculty iPads, respectively.

**Table 2:** Pathogen recovery from various selective media.

	Hospital location (n = 14)	Non-hospital location (n = 16)	*P* value
**Gram positive organisms**			
Coagulase negative Staphylococci broth, n (%)	14 (100)	15 (93.8)	1.000
Coagulase negative Staphylococci quantity, mean‡ ± SD	2.24±0.40	2.43±0.82	0.487
*S. aureus* broth, n (%)	10 (71.4)	12 (75)	1.000
*S. aureus* quantity, mean‡ (SD)	1.82±0.71	1.90±0.73	0.824
MRSA broth, n (%)	9 (64.3)	6 (37.5)	0.272
MRSA quantity, mean‡ ± SD	1.67±0.68	1.51±0.16	0.655
Enterococci broth, n (%)	14 (100)	16 (100)	–
Enterococci quantity, mean‡ ± SD	2.20±0.64	2.36±0.87	0.595
VRE broth, n (%)	1 (7.1)	0	0.467
VRE quantity, mean‡ ± SD	–	–	–
**Gram negative organisms**			
Mueller Hinton broth (non-selective), n (%)	14 (100)	16 (100)	–
Mueller Hinton quantity (non-selective), mean‡ ± SD	2.72±0.65	2.87±0.83	0.601
Levine broth, n (%)	7 (50)	8 (50)	1.000
Levine pink colonies*, mean‡ ± SD	1.59±0.59	1.86±0.74	0.430
Levine blue colonies#, mean‡ ± SD	2.23±0.83	1.3	–
MacConkey broth, n (%)	1 (7.1)	2 (12.5)	1.000
MacConkey LF (pink colonies), mean‡ ± SD	1.2	1.24±0.34	–
MacConkey NLF (colorless colonies), mean‡ ± SD	0	0	–
Cetrimide broth, n (%)	1 (7.1)	1 (6.3)	1.000
Cetrimide quantity, mean‡ ± SD	0.7	0.7	–

All quantities listed are in log CFU/mL organisms recovered from the respective selective media used to isolate the organisms as described in methods.

*Levine pink/colorless colonies identified non-lactose fermenting *Salmonella* spp., *Shigella* spp., and *Proteus* spp., as per package insert.

# Levine blue/black colonies identified *Enterobacter* spp. or *Klebsiella* spp., as per package insert.

‡ All means were only calculated for devices with organism growth.

## Discussion

Health information technology, which emphasises the use of electronic health records, has become commonplace in recent years [16]. Handheld ‘smart’ devices such as iPads, tablets, and smartphones are gaining popularity as technology in the healthcare system continues to advance and becomes increasingly wireless. While more recent data are emerging on the ability of tablet computers to act as fomites among healthcare workers in hospital, limited data are available which elucidate the presence of pathogens on tablet computers belonging to healthcare workers within outpatient practice settings [13],[14],[17]. Specifically, we sought to identify and compare organism colonization on iPads within a pharmacy school department where faculty members practice within a variety of healthcare settings.

Approximately 6 months following iPad distribution to faculty, the iPads were swabbed in order to elucidate the presence of select, clinically-relevant pathogens. We hypothesised that iPads used within hospital settings would have more frequent colonization with many of the nosocomial pathogens probed for in the study. While overall frequency of use did not differ between faculty in hospital settings compared to faculty in non-hospital settings, faculty in hospitals more frequently used their iPads in patient care areas (Table 1). We also noted that only 4 of 14 participants in the hospital group used their iPad daily in patient care areas and 9 of 14 used the device often in patient care areas (daily or weekly). This should be noted as a potential weakness of these data as not everybody in the hospital group used the device in a patient care area.

Interestingly, overall recovery of organisms and organism quantity did not differ between the groups (Table 2). Not surprisingly, common skin and gastrointestinal tract colonizers such as coagulase negative Staphylococci and Enterococci were commonly recovered from iPads indicating that the sources for most of the recovered organisms were the fingers of the user and not the environment in which the device was used. Only about half of the faculty in either group had cleaned their iPad at least once within the six months following their distribution. In 2013, Manning et al. published a set of common sense interventions for reducing contamination of mobile handheld devices [14]. These recommendations, termed the iPBundle, included (1) use of a waterproof barrier, (2) disinfection of the mobile device before and after the patient/family interface, (3) automatic reminders for regular disinfection, and (4) hand hygiene before and after use of a mobile device. While these recommendations are clearly useful, it remains unclear what the proper disinfection method should be in order to minimize damage to these devices while still properly sterilizing them. According to the Apple support website, the manufacturer recommends cleaning with a soft, slightly damp, lint-free cloth and advises against using window cleaners, household cleaners, aerosol sprays, solvents, alcohol, ammonia, or abrasives to clean iPad [18]. Kiedrowski et al. set out to answer this question and recently published a comparison of iPad disinfection methods.They noted that moist cloths, alcohol swabs, and bleach wipes were able to remove 100% of MRSA from iPad screens, however, there was no discussion of organism yield using their recovery method [13]. Howell et al. also aimed to answer this question and compared six different disinfectant wipes on iPads contaminated with MRSA, VRE, and *C. difficile* [17]. They concluded that Sani-Cloth CHG 2% (chlorhexidine 2%/alcohol 70%) wipes effectively disinfected the iPad against MRSA and VRE (but not *C. difficile*), with a residual antibacterial effect and without causing damage after 480 cleaning episodes. It should be noted, however, that the maximum duration of functionality was assessed after a 40-day period; therefore, it remains to be seen if this cleaning method could result in any long-term damage. Neither of these studies assessed sterilization of nosocomial Gram-negative pathogens, so it remains to be seen how effective these cleaning methods may be against the problematic Gram-negative species we found on iPads in our study. It should also be noted that Corning, a major supplier of glass for touch screen devices including tablet computers, announced the release of antimicrobial glass which may be used in future generations of touch screen devices [19]. However, it remains to be seen how effective the antimicrobial glass will be in reducing surface contamination and it will take time for all the previous generations of devices currently in use to be replaced.

Regardless of the location of the practice site, problematic nosocomial pathogens such as MRSA (64.3% and 37.5%), VRE (7.1% and 0%), and *P. aeruginosa* (7.1% and 6.3%) were recovered. This was less surprising for MRSA given that MRSA originating from outside healthcare institutions has become very common in the last 10 years with the rapid clonal expansion of community-associated MRSA, in particular USA300 *S. aureus* [20]. It is important to note that for both VRE and *P. aeruginosa*, the raw numbers of these organisms isolated (n = 1 for VRE and N = 2 for *P. aeruginosa* overall) was too low to draw any generalizable conclusion about the frequency of isolation of these pathogens. It should also be noted that confirmatory testing to definitively identify organisms isolated on selective agars were not performed for any pathogen. In addition, there are also important, potentially surface contaminating pathogens which were not screened for in our study, notably *Clostridium difficile*, which is known to form spores and survive on surfaces for extended periods of time. It is also unknown what the relative risk of infection from these devices is compared to other objects encountered in a healthcare setting.

In conclusion, a variety of nosocomial pathogens were isolated from iPads, regardless of the nature of the site in which they were used. Not surprisingly, the most commonly isolated organisms are normal human colonizers, though these organisms can be pathogenic. In addition, a variety of other pathogens, including antibiotic resistant pathogens, were also isolated from the iPads. Tablet computers can be reservoirs of pathogens and a means by which organisms can be moved from patient to patient within a variety of healthcare institutions and infection prevention strategies related to the devices should be employed.

## Supporting Information

Table S1 **Yield testing data for both Gram positive pathogens (** **tab 1** **) and Gram negative pathogens (** **tab 2** **).** Data is color coded by organism, then by iPad generation, and lastly by number of wet swabs used. Inoculum column is the concentration of organism suspension used in testing, “Amt applied” column is the raw number of bacteria applied to the surface of the iPad as determined by before and after weights of the swabs used to apply the suspensions.
(XLSX)
